# Evaluating the diagnostic performance of OpenBioLLM in neurology: A case-based assessment of a medical large language model

**DOI:** 10.1371/journal.pone.0332196

**Published:** 2025-09-25

**Authors:** Gholamreza Habibi, Shahryar Rajai Firouzabadi, Ida Mohammadi, Omid Kohandel Gargari

**Affiliations:** Farzan Artificial Intelligence Team, Farzan Clinical Research Institute, Tehran, Iran; UCSF: University of California San Francisco, UNITED STATES OF AMERICA

## Abstract

In the evolving field of neurological healthcare, deep learning technologies are gaining recognition for their potential to enhance diagnostic accuracy. Transformer-based models, particularly large language models (LLMs) such as OpenBioLLM, have shown promise in processing large datasets typical of neurological assessments. This study evaluates the diagnostic capabilities of OpenBioLLM in the realm of neurological conditions. The primary aim of this research is to assess the diagnostic accuracy, comprehensiveness, supplementation, and fluency of OpenBioLLM when applied to complex neurological case studies. Twenty-five complex neurology cases were selected from “Clinical Cases in Neurology.” OpenBioLLM was used to generate diagnoses and rationales for each case. Two independent medical doctors evaluated the responses based on accuracy, comprehensiveness, supplementation, and fluency, with discrepancies resolved by a third assessor. Statistical analyses included one-way ANOVA, Bartlett’s test, and Spearman’s rank correlation. OpenBioLLM achieved a mean accuracy score of 38%, a comprehensiveness score of 52%, a supplementation score of 24%, and a fluency score of 100%. The model could localize neurological lesions but often struggled with identifying the correct pathophysiological causes. Accuracy scores did not significantly vary by neurological disorder type. While OpenBioLLM shows potential in diagnosing neurological conditions, its performance metrics suggest it is not yet a reliable standalone tool. Future research should focus on fine-tuning the model and improving its reasoning capabilities to enhance diagnostic accuracy.

## Introduction

In the ever-evolving field of neurological healthcare, deep learning technologies are gaining recognition for their potential to enhance diagnostic accuracy, prognostication, and treatment approaches. These technologies are particularly vital for identifying complex patterns in the large datasets typical of neurological assessments [1,2]. Transformer-based models, a subset of large language models (LLMs), have garnered significant attention [3]. These models are proficient at handling substantial volumes of text data, enabling them to discern intricate relationships between words and generate contextually appropriate and coherent responses [4]. OpenAI’s Generative Pre-Trained Transformer (GPT) series represents the pinnacle of contemporary AI technology. The latest version, GPT-4, outperforms its predecessor, GPT-3.5, in interactive tasks, with marked improvements in various benchmark evaluations [5]. Among specialized large language models, OpenBioLLM has recently been introduced, specifically fine-tuned for medical tasks, and has been claimed to perform better than GPT-4 [6].

The performance of LLMs in neurological healthcare has been investigated by numerous studies, finding that LLMs can achieve passing grades on neurology board examinations [7,8], outperform neurologists on clinical diagnosis [9,10], and even detect [11] and localize [12] brain infarctions. A recent study evaluating ChatGPT-4’s diagnostic accuracy on 51 neurology cases found it produced similar diagnoses to human neurologists, which was backed by comprehensive reasoning on most cases [13], yet LLMs’ diagnostic performance on complex and challenging neurology cases remains underexplored. The application of LLMs in neurological practice also remains challenging due to a plethora of limitations and uncertainties [14]. These include faulty clinical reasoning and variable accuracy when presented with clinical scenarios that the LLMs have not been trained on [14]. To this end, specialized LLMs such as OpenBioLLM have been developed, which incorporate a diverse medical instruction dataset aimed at circumventing these limitations.

With this in mind, we aim to evaluate the diagnostic capabilities of OpenBioLLM in the realm of neurological conditions. By analyzing its performance on 25 complex neurology case studies, we seek to examine its performance in clinical diagnosis alongside an evaluation of its clinical reasoning.

## Methods

### Data source

Twenty-five complex neurology cases were selected from the book “Clinical Cases in Neurology,” each containing descriptions of signs, symptoms, relevant paraclinical findings, demographics, and important past medical histories [15]. The cases are a collection of common and uncommon neurological conditions collected over twenty years of clinical practice by Dr. Ondrej Dolezal at Dumfries and Galloway Royal Infirmary in Dumfries, United Kingdom. The diagnoses include Intra-Cranial Hemorrhages (n = 3), Brain tumors (n = 7), Neurodegenerative disorders (n = 3), Neuro-infections (n = 3), polyneuropathies (n = 2), and other disorders (n = 7).

### Study design

We utilized OpenBioLLM, sourced from the official Hugging Face repositories, and configured it according to the provided guidelines. The model was provided with each clinical case separately and was instructed to provide a diagnosis and rationale using the following prompt: “Please provide a list of differential diagnoses for this case and also write the most likely diagnosis and your reasoning.” The diagnosis presented as the most likely, and the reasoning behind it was used in our analyses. No case assistance was provided, and the model was not provided with any prior reference material to facilitate its diagnoses.

### Model evaluation

Performance outcomes and rating scales are similar to a previous study [16]. Briefly, two medical doctors independently evaluated the LLMs’ responses. Each response was assessed across four domains: accuracy, comprehensiveness, supplementation, and fluency. Discrepancies between the two evaluators were resolved through discussion with a third assessor so that only one assessment of the model’s performance was available for analysis. The inter-rater reliability for each outcome, however, is provided via Cohen’s Kappa values with κ > 0.7 considered acceptable. Accuracy and comprehensiveness were considered as the primary outcomes and rated on a three-point scale (0–2), while supplementation and fluency were secondary outcomes and were rated on a two-point scale (0–1). Primary outcomes were evaluated using the answers provided by the book’s author as a benchmark, while secondary outcomes lacked a control group and were assessed by each medical doctor based on their interpretation of the model’s answer.

### Primary outcomes

Accuracy was classified as inaccurate, partially accurate, or accurate. Inaccurate responses were those significantly different from the book’s answer, potentially leading to patient harm with no possibility of correct diagnosis, even if subsequent confirmation tests or imaging were conducted. Partially accurate responses correctly localized the lesion but identified an incorrect pathological cause or would lead to the correct diagnosis if subsequent confirmation tests were conducted (e.g., MRIs to rule out structural abnormalities). Accurate responses correctly localized the lesion(s) and identified the pathological cause, aligning with the book’s answer.

Comprehensiveness was rated as incomprehensive, partially comprehensive, or comprehensive. Incomprehensive responses lacked relevant reasoning for the diagnosis or provided irrelevant reasoning (e.g., symptoms not related to the diagnosis, signs inconsistent with diagnostic criteria). Partially comprehensive responses offered some relevant reasoning but failed to fully integrate all signs, symptoms, physical examination findings, and family history consistent with the diagnosis. Comprehensive responses provided relevant reasoning that encompassed all the provided information, consistent with the diagnosis in the case.

### Secondary outcomes

Supplementation referred to the inclusion of useful diagnostic information in the reasoning provided, which, while not required for a correct diagnosis, added valuable context (these included the patient’s age, habits, previous medical history, and/or profession). An example of this was OpenBioLLM using a previous medical history of spinal irradiation to reach a diagnosis of cervical myelopathy by arguing that this irradiation may be due to a pre-existing spondyloarthropathy that has accelerated degenerative changes in the cervical joints. Fluency was defined as the ability to comprehend the LLM’s response in one reading, characterized by the absence of repetitive statements and the presence of a cohesive structure throughout the answer.

### Statistical analysis

All analyses were conducted using Stata version 17.0 (StataCorp). The average scores on each outcome are reported as means and standard deviations or medians and interquartile ranges (IQR) according to normal distribution assessed via the Shapiro-Wilk test. For comparison of accuracy, comprehensiveness, and supplementation scores by diagnostic grouping, Kruskal-Wallis test was used. Inter-rater reliability was assessed via Cohen’s Kappa (κ) for each outcome separately. A κ > 0.7 was considered acceptable yet any disagreement between the two raters was resolved via a third rater so that only one assessment was available for analysis. A To assess the correlation between scores, Spearman’s rank correlation test was used, with a Spearman’s rho of >0.4 signifying moderate correlation and >0.6 a strong correlation. A p-value<0.05 was considered statistically significant.

## Results

### Accuracy

Of the 25 clinical cases, only 3 (12%) were diagnosed accurately, 13 (52%) were partially accurate, and 9 (36%) were inaccurate, ultimately resulting in a mean accuracy rate of 38% (Table 1). The correctly diagnosed cases consisted of amyotrophic lateral sclerosis (ALS) (n = 1), hereditary polyneuropathy (n = 1), and Guillain-Barré syndrome (n = 1). The partially accurate cases consisted of epidural hematoma (n = 1), spinal tumors (n = 1), brain tumors (n = 4), encephalitis (n = 1), chronic lymphomatous leukemia with CNS infiltration (n = 1), carotid artery dissection (n = 1), B12 deficiency (n = 1), meningitis (n = 1), hemosiderosis (n = 1), and lower spinal cord dual arteriovenous fistula (n = 1). The 9 undiagnosed cases included brain tumors (n = 3), hydrocephalus (n = 1), subdural hematoma (n = 1), multiple sclerosis (n = 2), mastoiditis (n = 1), and intra-parenchymal bleed (n = 1). The accuracy score of OpenBioLLM was 0.76 (SD: 0.66), which did not vary significantly by diagnostic grouping (p-value = 0.246) (Table 2). Overall, it appeared that AI models were capable of compiling neurological deficits and symptoms to identify the location of neurological lesions, yet were mostly incapable of diagnosing the correct cause and pathophysiology of the lesion (S1 Table). Inter rater reliability was acceptable (κ = 0.93).

**Table 1:** Number of accurate and comprehensive answers provided by OpenBioLLM.

Accuracy, N (%)		Comprehensiveness, N (%)	
Accurate	3 (12%)	Comprehensive	8 (32%)
Partially Accurate	13 (52%)	Partially Comprehensive	10 (40%)
Inaccurate	9 (36%)	Incomprehensive	7 (28%)

**Table 2:** Accuracy, comprehensiveness, supplementation, and fluency scores for OpenBioLLM.

	Accuracy score	P-value	Comprehensiveness score	P-value	Supplementation score	P-value	Fluency score
All cases	0.76 (0.66)		1.04 (0.79)		0 (0, 0)		1 (1, 1)
Intra-Cranial Hemorrhage (subdural hematoma + epidural hematoma + intra-parenchymal bleed) (n = 3)	0.34 (0.58)	0.246	0.34 (0.58)	0.432	0 (0, 0)	0.583	1 (1, 1)
Brain Tumors (n = 7)	0.57 (0.53)		1.14 (0.90)		0 (0, 1)		1 (1, 1)
NeuroDegenerative disorders (motor neuron disease + multiple sclerosis) (n = 3)	0.67 (1.16)		1 (1)		0 (0, 1)		1 (1, 1)
Neuro-Infections (encephalitis + meningitis + mastoiditis) (n = 3)	0.67 (0.58)		0.67 (0.58)		0 (0, 0)		1 (1, 1)
Polyneuropathy (n = 2)	2 (0)		2 (0)		0.5 (0, 1)		1 (1, 1)

Data are presented as mean (SD) or median (IQR).

### Comprehensiveness

Of the 25 proved answers, 8 (32%) were comprehensive, 10 (40%) were partially comprehensive, and 7 (28%) were incomprehensive, ultimately resulting in a mean comprehensiveness score of 52% (Table 1). The comprehensive answers were for cases of brain tumor (n = 3), ALS, hereditary polyneuropathy (n = 1), lower spinal cord dual arteriovenous fistula (n = 1), and Guillain-Barré syndrome (n = 1). Partially comprehensive answers comprised cases of epidural hematoma (n = 1), spinal tumor (n = 1), multiple sclerosis (n = 1), encephalitis (n = 1), brain tumors (n = 2), chronic lymphomatous leukemia with CNS infiltration, (n = 1), carotid artery dissection (n = 1), B12 deficiency (n = 1), meningitis (n = 1), and hemosiderosis (n = 1). Incomprehensive answers were for cases of multiple sclerosis (n-1), subdural hematoma (n = 1), mastoiditis (n = 1), brain tumor (n = 2), intra-parenchymal hemorrhage (n = 1), and hydrocephalus (n = 1). The comprehensiveness score of OpenBioLLM was 1.04 (SD: 0.79), which also did not vary significantly by diagnostic grouping (p-value = 0.432). inter rater reliability was acceptable (κ = 0.94). The comprehensiveness score of OpenBioLLM was strongly correlated with the accuracy score (Spearman’s rho = 0.843, p-value<0.001).

### Supplementation and fluency

**Fig 1:**
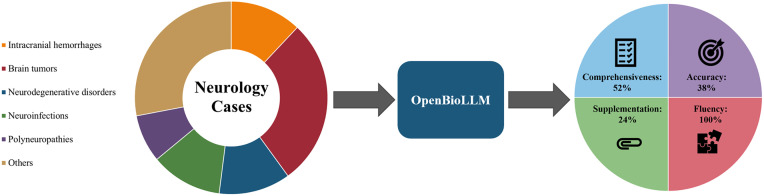
Study flowchart.

With regard to supplementary information, OpenBioLLM reached a mean supplementation rate of 24% and received a median score of 0 (IQR: 0, 0), with similar scores by diagnostic groupings (p-value = 0.583). Inter rater reliability was acceptable (κ = 0.88). The supplementation score was moderately correlated with accuracy (Spearman’s rho = 0.553, p-value = 0.004) as well as comprehensiveness (Spearman’s rho = 0.581, p-value = 0.002). Fluency was the best-performing domain of OpenBioLLM, where it achieved a mean fluency rate of 100% and a median score of 1 (IQR: 1, 1). There was no inter rater disagreement. A flowchart summarizing our findings is available in Fig 1. Table 3 presents Correlations matrix between accuracy, comprehensiveness, and supplementation of answers.

**Table 3:** Correlations matrix between accuracy, comprehensiveness, and supplementation of answers.

		Accuracy	Comprehensiveness	Supplementation
Accuracy	Spearman’s rho	1		
	P-value	.		
Comprehensiveness	Spearman’s rho	0.843	1	
	P-value	<0.001	.	
Supplementation	Spearman’s rho	0.553	0.581	1
	P-value	0.004	0.002	.

## Discussion

This study explored the capabilities of OpenBioLLM in diagnosing a variety of neurological conditions when presented with clinical neurologic vignettes. OpenBioLLM achieved a mean accuracy score of 38%, a mean comprehensiveness score of 52%, a mean supplementation score of 24%, and finally, a mean fluency score of 100%. We found that the comprehensiveness, supplementation, and fluency of the model were correlated with its accuracy. Additionally, none of the scores measuring the diagnostic abilities of OpenBioLLM varied significantly according to the type of neurological disorder.

With the emergence and recent popularity of LLMs, efforts have been made to investigate their accuracy in diagnosing a wide array of medical conditions [17], including neurological conditions. The most similar investigation to ours was conducted by Galetta et al., who gauged the diagnostic accuracy of GPT 4 when presented with 29 clinical vignettes of neurological conditions. GPT 4 presented the correct diagnosis only 52% of the time in its differential diagnoses, with the accurate answer listed as the leading diagnosis in only 24% of instances. Additionally, it was found that GPT 4 was able to localize the lesion 48% of the time, whereas in our study, OpenBioLLM localized the lesion in 64% of the cases [18]. In addition, Horiuchi et al. assessed ChatGPT-4’s diagnostic performance in neuroradiology by testing it on 100 published clinical cases from the American Journal of Neuroradiology. Using medical history and imaging findings, ChatGPT achieved an overall diagnostic accuracy of 50%. Accuracy did not significantly differ across anatomical regions (brain, spine, head & neck), but performance was notably poorer in brain cases involving CNS tumors (16%) compared to non-CNS tumors (62%) [19]. Furthermore, Koga et al. assessed the diagnostic capabilities of ChatGPT-3.5, ChatGPT-4, and Google Bard in predicting neuropathologic diagnoses from clinical case summaries of 25 patients with neurodegenerative disorders discussed at Mayo Clinic Clinico-Pathological Conferences. Each model generated multiple possible diagnoses with explanations, which were compared to expert-confirmed diagnoses. ChatGPT-4 correctly identified the primary diagnosis in 52% of cases—higher than ChatGPT-3.5 (32%) and Google Bard (40%). When considering whether the correct diagnosis was included anywhere in the differential, accuracy improved to 84% for ChatGPT-4 [10]. Furthermore, Brigo et al. evaluated ChatGPT-4.0’s ability to diagnose epilepsy using ILAE 2014 criteria and found very poor agreement with expert clinicians. The model had low sensitivity (17.6%) and missed a large proportion of true epilepsy cases, especially in older patients. While it showed better performance in ruling out epilepsy (specificity 81.4%), the overall diagnostic accuracy was insufficient for clinical application [20]. While most studies achieved relatively low rates of success, Cano-Besquet et al., evaluating the diagnostic accuracy of ChatGPT-4, found that it achieved a comprehensive diagnostic success rate of 96.1%, comparable to that of consultant neurologists (94.1%). While neurologists were more likely to provide highly comprehensive diagnoses, integrating ChatGPT-4’s input with that of consultants led to complete diagnostic coverage. Yet their findings may reflect structured input and expert confirmation, which likely minimized model errors [13].

As evident by this report and in accordance with the literature, LLMs seem to fare worse when attempting to diagnose cases of neurological conditions. A preprint meta-analysis comparing the diagnostic accuracy of generative AI and physicians has shown that there is a decline of 21.7% in the diagnostic accuracy of LLMs when presented with cases of neurological conditions versus general medicine [21]. Interestingly, a similar pattern is observed among the learners of neurological sciences, with many perceiving neurology as one of the most challenging components of the medical curriculum. This issue was dubbed ‘neurophobia’ by Jozefowicz in 1994 [22], and neuroanatomy and lesion localization have been found as key culprits contributing to it [23]. A similar condition appears to plague LLMs; however, it is unclear what factors contribute to it, as we and Galetta et al. have noted that the ability of LLMs to pinpoint the location of lesions did not correlate with their ability to provide accurate diagnoses. In our experience, one of the main problems in the provided diagnoses was that the model often disregarded the patient’s baseline information, such as age, gender, and comorbidities, and would provide diagnoses that were uncommon for that demographic group. For example, the model would diagnose elderly patients experiencing new-onset seizures with primary or genetic epilepsy, even though new-onset seizures in old age occur mainly as a consequence of accumulated injuries to the brain and are often secondary to cardiovascular, degenerative, oncologic, and traumatic causes [24]. Future research is needed to analyze the flaws of the logical process through which the LLMs make diagnoses so that areas of weakness can be identified and targeted by fine-tuning.

Regarding comprehensiveness and providing supplementary reasoning, there seems to be little research into the clinical reasoning of LLMs when diagnosing neurological conditions. The reports previously described have all investigated the accuracy of LLMs, but the chain of thought leading to the diagnoses has been paid little mind. Nevertheless, it is crucial to prompt the LLMs to provide reasoning for their diagnosis in order to assess for any blind spots or mistakes. For example, when Chen et al. assessed the clinical reasoning of ChatGPT for whether or not to pursue thrombectomy in stroke patients, it was shown that while ChatGPT agreed with the physician’s decision in 54.3% of the cases, it had made errors in its reasoning in 8.8% of the cases, consisting of mathematics, logic, and misinterpretation errors [25]. In addition, it should be noted that we found a significant correlation between accuracy and the comprehensiveness and supplementation of the reasoning. One explanation could be that prompting the model to provide explanations for the diagnoses may increase its accuracy [26]. Future studies are needed so that the reasoning of LLMs and their possible relationship with the accuracy of the model can be explored.

Although we included a limited number of studies in each diagnostic grouping to be able to adequately assess the model’s performance in each grouping, this preliminary analysis allowed us to identify the model’s potential biases for certain diagnostic groupings. In particular, OpenBioLLM appears to struggle more when presented with cases of intracranial hemorrhages. We recommend future iterations of the LLM to pay closer attention to this particular diagnosis and perhaps train their model on more cases of this diagnostic grouping.

Given OpenBioLLM’s limitations in diagnostic accuracy and reasoning, it is important to consider methods that could enhance its clinical performance. Retrieval-Augmented Generation (RAG) is a promising approach that combines language generation with real-time information retrieval from external sources. Unlike standard LLMs, RAG can access and integrate relevant medical knowledge on demand, improving the accuracy and contextual relevance of its outputs. RAG is potentially useful for this type of study, as it could help address the model’s struggles with incorporating patient-specific factors and generating well-supported diagnoses in complex neurological cases [27].

### Limitations

We assessed OpenBioLLM on a small number of diverse and complex clinical vignettes, and to achieve more precise estimations of the model’s performance, it is recommended that the model be tested on a larger number of cases. Our selection of these cases was not representative of the full spectrum of diseases observed in clinical practice [28,29], with a gross underrepresentation of cerebrovascular accidents in our study. These twenty-five cases, however, were selected due to being more challenging compared to standard neurological cases and represented clinical scenarios that may be more uncommon yet are considered essential education material for neurologists [15]. We did not compare the model to other LLMs, such as GPT, in this study. This comparison is recommended in future research as it can provide a better understanding of the level of the model’s performance and whether the development of LLMs specific to clinical practice generates significantly more accurate answers. The performance outcomes used in our study, while similar to some studies [13,16], differ from some previous research due to a lack of a standardized assessment and classification system [17]. The rating scales used in our study are also similar to a previous work [16] yet there is also no consensus on rating scales [17]. Lastly, secondary outcomes in our study were not comapred to a control group or a benchmark for evaluation, which may introduce bias into our reported evaluations. Yet, these findings can help future studies identify which LLMs can serve as a comparator or control group in their studies and whether one LLM can be a potential gold standard.

## Conclusions

While OpenBioLLM shows potential in diagnosing neurological conditions, its current performance metrics make it a less-than-ideal standalone tool for performing this task. Future research should assess this model using a larger number of cases. In addition, fine-tuning and prompting the model to provide more of its reasoning can be areas that can be explored to increase its accuracy.

## Supporting information

S1 Table OpenBioLLM diagnoses for each corresponding case. (DOCX)
