# Supplementary material for: Evaluating the diagnostic performance of OpenBioLLM in neurology: A case-based assessment of a medical large language model
Source: PLoS One. 2025 Sep 25;20(9):e0332196. doi: 10.1371/journal.pone.0332196 (PMC12463255; doi:10.1371/journal.pone.0332196)
Supplement: S1 Table — (DOCX) [file pone.0332196.s001.docx]

Table S1: OpenBioLLM diagnoses for each corresponding case.

| Case | OpenBioLLM diagnosis |
| --- | --- |
| Case 1  Cervical cord compression and myelopathy due to epidural hematoma | Subdural hematoma (partially accurate) (partially comprehensive) (fluent) |
| Case 2  Multiple sclerosis with lesions in the pons close to trigeminal nerve | Trigeminal neuralgia (inaccurate) (incomprehensive) (fluent) |
| Case 3  Subacute chronic subdural hematoma in the left hemisphere | Cervical myelopathy (inaccurate) (incomprehensive) (fluent) |
| Case 4  Spinal cord compression at thoracic level due to meningioma | Cervical myelopathy (partially accurate) (partially comprehensive) (fluent) (supplementation) |
| Case 5  Multiple sclerosis with lesions involving right lateral thalamus | Syringomyelia (Inaccurate) (partially comprehensive) (fluent) |
| Case 6  Meningioma in right medial fossa with adjacent compression and edema | Complex partial seizures (partially accurate) (comprehensive) (supplementation) (fluent) |
| Case 7  Left mastoiditis with adjacent left temporal lobe swelling | Temporal lobe epilepsy (inaccurate) (incomprehensive) (fluent) |
| Case 8  Temporal hemorrhagic encephalitis | Cerebral abscess (partially accurate) (partially comprehensive) (fluent) |
| Case 9  Left middle cranial fossa meningioma with adjacent edema and compression of left lateral ventricle | Temporal lobe epilepsy (partially accurate) (partially comprehensive) (fluent) |
| Case 10  Left temporal lobe glioma with compression of left lateral ventricle | Psychogenic non-epileptic seizure (inaccurate) (incomprehensive) (fluent) |
| Case 11  Left frontal Intraparenchymal bleed | Third cranial nerve palsy (inaccurate) (incomprehensive) (fluent) |
| Case 12  Posterior medullary glioma with extension into foramen Magendie | Epilepsy (inaccurate) (incomprehensive) (fluent) |
| Case 13  Chronic lymphocytic leukemia with CNS infiltration in the midbrain, pons, medulla, epiconus, and conus medullaris | Progressive multifocal leukoencephalopathy (partially accurate) (partially comprehensive) (fluent) |
| Case 14  Obstructive hydrocephalus due to third ventricle colloid cyst blocking foramen Monro | Autonomic dysfunction (inaccurate) (incomprehensive) (fluent) |
| Case 15  Bilateral carotid artery dissection with adjacent hypoglossal nerve involvement | Brainstem stroke (partially accurate) (partially comprehensive) (fluent) |
| Case 16  ALS with internal capsule and cerebellar peduncle involvement | Motor neuron disease (accurate) (comprehensive)  (supplementation) (fluent) |
| Case 17  B12 deficiency with cervical spinal cord involvement | Peripheral neuropathy (partially accurate) (partially comprehensive) (fluent) |
| Case 18  Meningitis with associated radiculitis (cervical root involvement) | Cervical radiculopathy (partially accurate) (partially comprehensive) (fluent) |
| Case 19  Nasopharyngeal tumor with extension into cavernous sinus and left temporal lobe with compression of trigeminal nerve | Vestibular Schwannoma (partially accurate) (comprehensive)  (supplementation) (fluent) |
| Case 20  Superficial hemosiderosis of brain and cervical spine | Motor neuron disease (partially accurate) (partially comprehensive) (fluent) |
| Case 21  Hereditary polyneuropathy | Hereditary neuropathy (accurate) (comprehensive) (fluent) |
| Case 22  Vestibular schwannoma in the left pontocerebellar angle with damage to the 8^th^ nerve | Brainstem stroke (partially accurate) (comprehensive) (fluent) |
| Case 23  Tumor in foramen magnum extending into the jugular foramen and condylar canal | Multiple System Atrophy (inaccurate) (partially incomprehensive) (fluent) |
| Case 24  Lower spinal cord dual arteriovenous fistula | Spinal stenosis (partially accurate) (comprehensive)  (supplementation) (fluent) |
| Case 25  Guillain-Barré Syndrome | Guillain-Barré Syndrome (accurate) (comprehensive)  (supplementation) (fluent) |
